# Cardiopulmonary Resuscitation in an Average Brazilian Intensive Care Unit: Should We Perform Less or Better?

**DOI:** 10.21470/1678-9741-2017-0036

**Published:** 2017

**Authors:** Leonardo Augusto Miana, Marcella Mendes Moraes, Bernardo Mendes Moraes, Pedro Guilherme Ponte, Eduardo Venturelli Júnior, Rodrigo Urbano Mallosto, Alexander Moreira-Almeida

**Affiliations:** 1 Faculdade de Medicina da Universidade Federal de Juiz de Fora (FAMED-UFJF), Juiz de Fora, MG, Brazil.; 2 Instituto do Coração do Hospital das Clínicas da Faculdade de Medicina da Universidade de São Paulo (InCor HC-FMUSP), São Paulo, SP, Brazil.

**Keywords:** Hypothermia, Heart Arrest, Induced Shock

## Abstract

**Introduction:**

Few data can be found about cardiac arrest in the intensive care unit outside reference centers in third world countries.

**Objective:**

To study epidemiology and prognostic factors associated with cardiac arrest in the intensive care unit (ICU) in an average Brazilian center.

**Methods:**

Between June 2011 and July 2014, 302 cases of cardiac arrest in the intensive care unit were prospectively evaluated in 273 patients (age: 68.9 ± 15 years) admitted in three mixed units. Data regarding cardiac arrest and cardiopulmonary resuscitation were collected in an "Utstein style" form and epidemiologic data was prospectively obtained. Factors associated with do not resuscitate orders, return of spontaneous circulation and survival were studied using binary logistic regression. Statistical package software used was SPSS 19.0 (IBM Inc., USA).

**Results:**

Among 302 cardiac arrests, 230 (76.3%) had their initial rhythm recorded and 141 (61.3%) was in asystole, 62 (27%) in pulseless electric activity (PEA) and 27 had a shockable rhythm (11.7%). In 109 (36.1%) cases, cardiac arrest had a suspected reversible cause. Most frequent suspected cardiac arrest causes were hypotension (n=98; 32.5%), multiple (19.2%) and hypoxemia (17.5%). Sixty (19.9%) cardiac arrests had do not resuscitate orders. Prior left ventricle dysfunction was the only predictor of do not resuscitate order (OR: 3.1 [CI=1.03-9.4]; *P*=0.04). Among patients that received cardiopulmonary resuscitation, 59 (24.4%) achieved return of spontaneous circulation and 12 survived to discharge (5.6%). Initial shockable rhythm was the only return of spontaneous circulation predictor (OR: 24.9 (2.4-257); *P*=0.007) and survival (OR: 4.6 (1.4-15); *P*=0.01).

**Conclusion:**

Cardiopulmonary resuscitation rate was high considering ICU patients, so was mortality. Prior left ventricular dysfunction was a predictor of do not resuscitate order. Initial shockable rhythm was a predictor of return of spontaneous circulation and survival.

**Table:** 

Abbreviations, acronyms & symbols	
CA	= Cardiac arrest
CPR	= Cardiopulmonary resuscitation
CPS	= Cerebral Performance Scale
DNR	= Do no resuscitate order
ICU	= Intensive care unit
ILCOR	= International Liaison
IQR	= Interquartile range
PEA	= Pulseless electric activity
PetCO_2_	= Partial pressure of end-tidal carbon dioxide
ROSC	= Return of spontaneous circulation
VT/VF	= Ventricular Tachycardia/ Ventricular Fibrillation

## INTRODUCTION

Cardiac arrest (CA) is a public health issue. It is assumed that 100000 in-hospital CA take place in Brazil every year^[1]^. According to the last available public data (DATASUS), in 2013, 120.000 in-hospital deaths were resisted in Minas Gerais state, and approximately 8.000 only in Juiz de Fora^[2]^. It is known that 50% of in-hospital death takes place in Intensive Care Units (ICU)^[3]^. Despite increasing complexity in cases, survival rate after in-hospital CA has been improving in North America^[3]^.

Since 1992, the International Liaison (ILCOR) has been working on providing evidence-based guidelines in Cardiopulmonary Resuscitation (CPR)^[4]^. In 2013, Brazilian researchers developed a local adaptation of these guidelines in order to improve adherence in Portuguese speaking physicians^[1]^.

The volume of studies on the subject and the registration of cases attended, either in-hospital or extra-hospital cardiac arrest, have grown expressively^[5,6]^. In several countries, there is a national register of attendance of CPRs^[3,6,7]^. This record makes it possible to evaluate the results, study the characteristics and epidemiology of each site, identify the main problems and propose improvements, increasing the survival of patients^[3,6]^.

There has been an exponential growing number of publications regarding CPR records, including out-of-hospital and in-hospital cases^[5,6]^. Many countries developed an CPR National Registry^[6,7]^. These records make it possible to evaluate the results, characteristics and epidemiology of each site, identify the main problems and propose improvements, increasing patients survival^[3,6]^.

Nevertheless, there is no registry in Brazil, so far^[1]^. There is lack in data regarding ICU-CPR profile and prognostic factors associated with death or survival. There are few Brazilian studies and almost all of them were developed in reference university centers, that may not represent real-world concerning CPR in Brazilian ICUs^[8,9]^. This study prospectively investigated demographic profile and prognostic factors in ICU-CA and CPR in three mixed units is an average Brazilian city.

## METHODS

This study was approved by the local Ethics Committee under the number 0120.0.420.000-10.

Between June 2011 and July 2014, 302 CA were observed in 273 patients in three mixed ICUs, with a total of 30 ICU beds.

Variables concerning CPR were registered in a Utstein style sheet form^[10]^. Demographic data were collected prospectively. CPR survivors were followed until hospital discharge and neurological status evaluated using Cerebral Performance Scale (CPS)^[11]^.

Studied variable included age, sex, ICU admission diagnosis, CA cause, event time, initial rhythm, do no resuscitate order (DNR), medications and dosing during CPR, defibrillation, previous diagnosis, previous intra-arterial monitoring, previous vasoactive-inotrope usage, left ventricular ejection fraction, CPR duration, return of spontaneous circulation (ROSC), therapeutic hypothermia, hospital discharge and CPS.

Primary end-point was survival to hospital discharge and secondary end-points were ROSC and DNR. ROSC was considered only if pulse was present after one hour after CPR cessation and no need to further CPR during this period.

Average patient's age was 68.8±14.9 years (19-99 years). Male sex was preponderant (51.6%). ICU admission diagnoses are listed in Table 1.

**Table 1:** ICU admission diagnostic list.

Diagnosis on ICU admission	Patients (n)	%
Postoperative care of cardiac surgery	76	27.8
Cardiologic cause	50	18.3
Non-cardiac surgery	40	14.7
Neurologic cause	26	9.5
Sepsis	30	11
Pulmonary cause	23	8.4
Others	26	9.5
Non-specified	2	0.8

### Statistical Analysis

Kolmorov-Smirnov and Shapiro-Wilkis were used as normality tests. Continuous data with normal distribution were described as average and standard deviation. For a non-normal distribution, median and interquartile range (IQR) were used. Nominal and ordinal data were presented as percentages. Exact Fisher test or Mann-Whitney were chosen for group comparisons. Binary logistic regression analyzed prognostic factors for the end-points. A *P* value of 5% was used.

## RESULTS

Demographic data of the 302 ICUCA studied are shown in Table 2.

**Table 2:** ICU-CA demographic data (n=302).

CA suspected cause according to attending physician	Hypotension	98 (32.4%)
	Multiple Causes	58 (19.2%)
	Hypoxemia	53 (17.6%)
	Metabolic Acidosis	25 (8.3%)
	MI and cardiogenic shock	25 (8.3%)
	Others	17 (5.6%)
	Non-specified	26 (8.6%)
CA initial rhythm	Asystole	141 (46.7%)
	PEA	62 (20.5%)
	VT/VF	27 (9%)
	Non specified	72 (23.8%)
Oral intubation	Intubated	285 (94.4%)
	Extubated	14 (4.6%)
	Non-specified	3 (1%)
Intra-arterial pressure Monitoring	Yes	207 (68.5%)
	No	95 (31.5%)
Vasoative-inotropic	Vasodilation	4 (1.3%)
	Inotropes	8 (2.7%)
	Vasopressors	181 (60%)
	2 or + drugs	53 (17.5%)
	None	49 (16.2%)
	Non-specified	7 (2.3%)
ICU	ICU 1	224 (74.2%)
	ICU 2	45 (14.9%)
	ICU 3	33 (10.9%)
CA time of day	Day (7am-7pm)	95 (31.4%)
	Night (7pm-midnight)	35 (11.6%)
	Dawn (midnight-7am)	47 (15.6%)
	Non-specified	125 (41.4%)

CA=cardiac arrest; MI=myocardial infarction; PES=pulseless electrical activity; VT/VF=ventricular tachycardia/ventricular fibrillation; ICU=intensive care unit

DNR was applicable in 60 (19.9%) patients. Univariate analysis found that prior left ventricular dysfunction, coronary disease, stroke as admission diagnosis, vasoactive drugs infusion, intra-arterial pressure monitoring and arrest during the dawn were associated with more DNR, while sepsis as an admission diagnosis was less associated with DNR (Table 3). However, multivariate analysis identified prior left ventricular dysfunction as a predictor of DNR (OR=3.1 [CI=1.03-9.4]; *P*=0.045) (Table 3).

**Table 3:** Univariate and Multivariate analysis regarding DNR order (n=302).

Variable	Univariate		Multivariate	
	OR (CI 95%)	*P* value	OR (CI 95%)	*P* value
Male sex	1.03 (0.5-1.8)	0.91		
Age	1.0 (0.9-1.0)	0.48		
Suspected reversible CA cause	1.4 (0.7-2.6)	0.27		
Non-shockable initial rhythm	3.5 (0.8-15.6)	0.09		
Extubated	0.4 (0.1-2.1)	0.48		
Intra-arterial pressure monitoring	2.0 (1.1-3.7)	0.01	1.03 (0.3-3.2)	0.9
Vasoactive infusion	3.3 (1.3-8.8)	0.01	6.5 (0.7-62.9)	0.1
Dawn CA time	2.1 (1.1-4.2)	0.03	2.02 (0.7-6.03)	0.2
Stroke	2.6 (0.9-7.3)	0.08	0.9 (0.06-13.7)	0.9
Previous CAD	1.8 (1.01-3.2)	0.04	0.8 (0.3-2.5)	0.7
Postoperative care of cardiac surgery	1.2 (0.6-2.2)	0.6		
Sepsis	0.4 (0.2-0.7)	0.003	0.6 (0.2-1.8)	0.3
Prior Left Ventricular Dysfunction	3.1 (1.4-6.6)	0.003	3.1 (1.03-9.4)	0.045

CA=cardiac arrest; CAD=coronary artery disease

There was 242 CPR in 213 patients. Twenty-five patients were resuscitated twice, one received three CPR and another received four. ROSC was achieved in 59 (24.4%) cases. Older age and CA in the dawn (between 0 and 7 am) were associated with less ROSC as investigated by univariate analysis (Table 4). CA with initial shockable rhythm had a 68% ROSC rate, while asystole (18.3%), PEA (37.2%) and non-identified rhythms (8.5%) were associated with significantly less ROSC rate (OR: 24.9 (CI=2.4-257); *P*=0.007) (Table 4).

**Table 4:** Univariate and Multivariate analysis of variables related to ROSC (n=242).

Variable	Univariate		Multivariate	
	OR (95% CI)	*P* value	OR (95% CI)	*P* value
Male sex	0.7 (0.4-1.4)	0.4		
Age	0.9 (0.96-0.99)	0.03	0.9 (0.9-1.1)	0.7
Reversible cause	1.2 (0.7-2.4)	0.4		
Shockable rhythm	6.9 (2.8-17.4)	0.0001	24.9 (2.4-257)	0.007
Intubation	0.7 (0.3-2.1)	0.5		
Intra-arterial pressure monitoring	1.9 (0.9-3.7)	0.06	1.8 (0.3-11.9)	0.6
Vasoactive drugs infusion	0.6 (0.3-1.3)	0.2		
CA time between 7am-midnight	2.2 (1.05-4.7)	0.03	1.5 (0.3-8.1)	0.6
Time before first defibrillation	1.03 (0.9-1.1)	0.6		
Total defibrillation charge (J)	0.9 (0.8-1.1)	0.2		
Time before intubation	0.9 (0.8-1.1)	0.4		
Time before epinephrine	1.03 (0.8-1.3)	0.8		
Epinephrine as first medication	2.7 (0.5-13.5)	0.2		
Stroke	0.7 (0.2-3.7)	0.7		
CAD	1.2 (0.7-2.3)	0.5		
Postoperative care of cardiac surgery	1.8 (0.9-3.3)	0.07	1.3 (0.2-7.7)	0.8
Sepsis	0.5 (0,27-1,2)	0.1		
Normal Left Ventricular function	3.2 (0.9-11.6)	0.08	4.7 (0.6-39.7)	0.15
LV Ejection Fraction	1.0 (0.99-1.05)	0.1		

CA=cardiac arrest; CAD=coronary artery disease; LV=left ventricle

When CPR was analyzed regarding response time, it was noticed a median time of 2 minutes between beginning of CPR and delivery of first defibrillation. Meanwhile, median time to intubation and first epinephrine dose was 7.5 minutes and 1 minute, respectively.

**Fig. 1:**
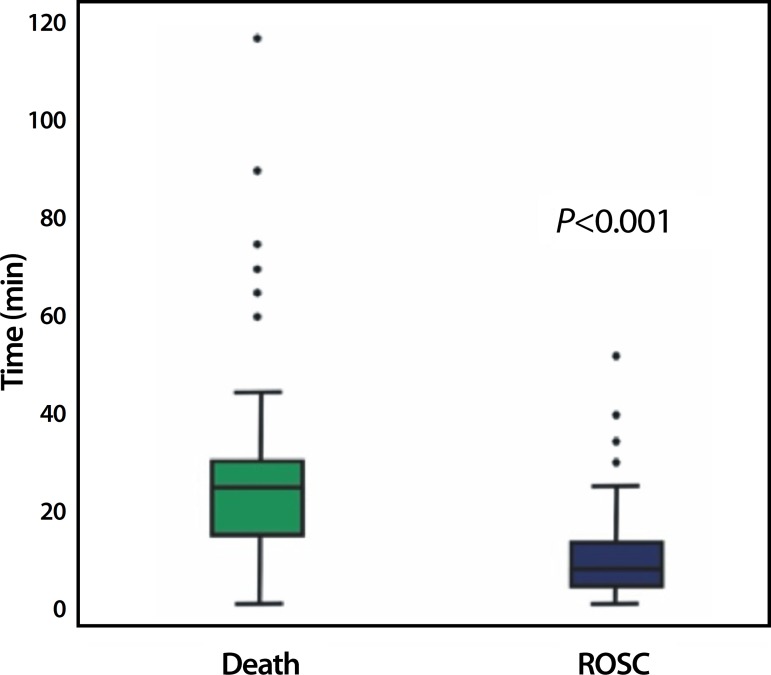
Comparison between ROSC CPR cases and unsuccessful CPR time. ROSC=return of spontaneous circulation, *P<0.0001 Mann-Whitney test.

CPR time varied from one minute to two hours. Median CPR time was 19.5 minutes (IQR=7-30 min). Median CPR time in ROSC cases was 8 minutes (IQR=4-14 min), while in unsuccessful resuscitations it was 25 minutes (IQR=15-30 minutes; *P*<0.001; Figure 1). All CPRs timetable is represented in Table 5. Twenty-seven patients exhibited shockable rhythms. Fifteen of them received one defibrillation, three were defibrillated twice and eight received three or more defibrillations.

**Table 5:** CPR timetable.

CPR duration (min)	N (%)	Cumulative (%)
1-3	13 (12.7)	12.7
4-10	29 (28.5)	41.2
11-20	21 (20.6)	61.8
21-30	23 (22.5)	84.3
31-40	6 (5.9)	90.2
41-60	4 (3.9)	94.1
>60	6 (5.9)	100
Total	102 (100)	

CPR=cardiopulmonary resuscitation

Median epinephrine total dose was 4 mg (IQR=2-8 mg). Dosing distribution is illustrated in Table 6.

**Table 6:** Epinephrine dosing distribution.

Epinefrin dosage (mg)	N (%)	Cumulative (%)
1	10 (13.5)	13.5
2	9 (12.2)	25.7
3	12 (16.2)	41.9
4	10 (13.5)	55.4
5-8	21 (28.4)	83.8
> 8	12 (16.2)	100
Total	74 (100)	

mg=miligram(s)

Other medications administered during CPR were atropine in 31 (13%) cases, sodium bicarbonate in 5.9% (14 cases), amiodarone in 10 (4.2%), norepinephrine in 7 (2.9%) cases, bolus saline infusion in 6 (2.5%), calcium gluconate in 1.7% (4 cases) and lidocaine and 50% glucose in one case each.

E-CPR was attempted in one patient. None of the patients were submitted to therapeutic hypothermia.

Survival to discharge was achieved in 12 patients out of 213 (5.6%). Ventricular tachycardia/ventricular fibrillation showed a 4.9 odds ratio when compared to other initial rhythms. No other predictor of survival was detected (Table 7)

**Table 7:** Univariate and multivariate analysis of variables related to survival (n=213).

Variable	Univariate		Multivariate	
	OR (95% CI)	*P* value	OR (95% CI)	*P* value
Male sex	1.7 (0.5-5.1)	0.4		
Age	0.97 (0.93-1.01)	0.1		
Reversible cause	1.02 (0.3-3.1)	0.9		
Shockable initial rhythm	4.9 (1.5-15.9)	0.008	4.6 (1.4-15)	0.01
Previous intubation	0.8 (0.1-6.7)	0.8		
Intra-arterial pressure monitoring	1.6 (0.5-5.2)	0.4		
Vasoactive drugs infusion	0.4 (0.1-1.5)	0.2		
CA time between 7am-midnight	1.04 (0.3-3.5)	0.9		
Time before first defibrillation	1.03 (0.8-1.3)	0.8		
Total defibrilation charge (J)	1.0 (0.99-1.01)	0.3		
Time before intubation	1.02 (0.9-1.2)	0.8		
Time before epinephrine	2.2 (0.3-15)	0.4		
Epinephrine as first medication	1.4 (0.2-10.2)	0.7		
Stroke	0.3 (0.03-3.5)	0.4		
CAD	1.3 (0.4-4.2)	0.6		
Postoperative care of cardiac surgery	1.3 (0.4-4.2)	0.6		
Sepsis	0.18(0.02-1.38)	0.1		
Normal left ventricular function	1.5 (0.15-14.7)	0.7		
LV ejection fraction	1.0 (0.9-1.06)	0.9		
CPR time	0.98 (0.93-1.04)	0.5		

CA=cardiac arrest; CAD=coronary artery disease; LV=left ventricle

Out of 12 survival patients, 10 presented a CPS score between 1 and 2, while other two presented a CPS of 3. One of these was a spine trauma patient with paraplegia before CA.

## DISCUSSION

This data represents a three-year registry in three ICU of an average sized city in Brazil. This analysis presents some relevant issues regarding ICU-CA in developing countries. It also allows some inferences about CPR delivered quality.

It is known that ICU-CA survival ranges between 0-42%, depending on many factors, such as team experience and training and patients complexity/profile^[6]^.

Recent studies pointed out increasing survival over the last years, especially with widespread adoption of ILCOR Guidelines^[1,3,4,7,12]^.

The present study noticed a DNR rate around 20% in this population. Since DNR orders in Brazil are not routinely adopted and vary greatly between hospitals and physicians, we evaluated variables related to this decision.

Previous Brazilian data report 65% DNR rate in ICU patients^[13]^. Previous international studies report a rate of DNR among inhospital and ICU-CA between 60% and 95%^[6,13-15]^.

In developed countries, mainly United States, there is a clear concern among family and ICU team about DNR orders in poor prognostic patients^[16,17]^. As abovementioned, neither the participating ICUs nor the Brazilian hospitals had an expressed DNR order for this type of patients, so the decision of beginning CPR depended primarily on the physician on duty.

As expected and reported previously^[6,13-15]^, variables such as age, admission diagnosis, cause of CA and previous vasoactive drug administration were related to DNR orders in our population, yet they were not independent predictors. The only independent predictor in our series was left ventricular dysfunction. Although it is not a common finding, it may represent the sickest patients in this sample.

CPR maneuvers in very sick patients with low or no prognosis is a very disputable topic and concern about it is gaining space in the literature^[18,19]^. Guidelines and current data assure the importance of prevention CA and selecting more and more which cases to perform CPR^[18,19]^.

In the present study, our CA prevalence was 75 cases per 1000 ICU admissions, which is high compared to other international reports that relate 13 to 29 CA per 1000 ICU admissions^[5-7]^.

Only one out of four CPR patients in our series have achieved ROSC. This result is similar to one previously reported Brazilian experience^[8]^, but very poor compared to current literature, including other Brazilian reports^[9,20]^.

Because of that, survival rate was low (5.6%) when compared to international literature^[3,5-7,13-15]^. It is clear that there was a high rate of CPR in the ICU (low DNR orders) and a bad result compared to the literature. Were there too many attempts of resuscitation?

When the aspects of CPR were analyzed, ventricular tachycardia/ ventricular fibrillation (VT/VF) was found to be the only predictor of ROSC. That finding is very similar to previous reports^[5,19]^. Other findings, such as CA immediate causes, admission diagnosis e resuscitation timing were not divergent from the literature^[8,9,19-21]^.

Some other observations lead us to infer that there was not a complete adherence to the guidelines. At least 31 cases received atropine and none of the patients that achieved ROSC were submitted to hypothermia. At the time of the study, hypothermia was suggested by the guidelines, especially in VT/VF cases^[22,23]^. Although some experimental studies suggest that partial pressure of end-tidal carbon dioxide (PetCO_2_) may not predict ROSC as expected^[24]^, its use is encouraged by the guidelines^[1]^. Yet its use was not reported in any of the 302 cases.

The ICU admission diagnosis table revealed that over 40% of the patients were surgical cases, known to be patients with better prognosis^[6,12]^. Mechanical circulatory support, which may positively impact survival in these patients^[25]^, was not available in those ICUs.

On the other hand, average age was 70 years-old with a high incidence of vasoactive drugs administration and the main immediate cause of CA was hypotension, leading to the idea that it was a high-risk cohort.

In 2003, a United States registry of 15k CPRs showed that arrhythmias and hypoxia (reversible causes) were the main immediate CA causes. Hypotension was in third. ROSC and survival rate was 44 and 17%, respectively^[26]^. Recently, the same registry, now with over 80k patients points out a tendency to increase the survival rate (22.3%)^[3]^.

In Brazil, the adoption of a National Registry and data bank that might allow results evaluation and comparison would be very desirable and may eventually save thousands of lives.

## CONCLUSION

The prospective evaluation of 302 CA in three years in three different ICUs identified 24.4% and 5.6% ROSC and survival rates, respectively. Shockable rhythm was the only predictor of ROSC and survival.

There was a 19.9% rate of DNR orders in the ICU-CA and left ventricular dysfunction was a predictor of DNR.

**Table:** 

Authors' roles & responsibilities	
LAM	Concept/design; drafting article; data interpretation; final approval of the version to be published
MMM	Data collection; data interpretation; final approval of the version to be published; final approval of the version to be published
BMM	Data collection; data interpretation; data collection; data interpretation, Statistics; final approval of the version to be published
PGP	Data collection; data interpretation; statistics; final approval of the version to be published
EVJ	Data collection; data interpretation; final approval of the version to be published
RUM	Data collection; data interpretation; final approval of the version to be published
AMA	Concept/design; drafting article; data interpretation; final approval of the version to be published
